# Reliable Execution Based on CPN and Skyline Optimization for Web Service Composition

**DOI:** 10.1155/2013/729769

**Published:** 2013-07-07

**Authors:** Liping Chen, Weitao Ha, Guojun Zhang

**Affiliations:** ^1^College of Mathematics and Information Science, Network Engineering Technology Center, Weinan Normal University, Weinan 714000, China; ^2^College of Communication Engineering, Network Engineering Technology Center, Weinan Normal University, Weinan 714000, China

## Abstract

With development of SOA, the complex problem can be solved by combining available individual services and ordering them to best suit user's requirements. Web services composition is widely used in business environment. With the features of inherent autonomy and heterogeneity for component web services, it is difficult to predict the behavior of the overall composite service. Therefore, transactional properties and nonfunctional quality of service (QoS) properties are crucial for selecting the web services to take part in the composition. Transactional properties ensure reliability of composite Web service, and QoS properties can identify the best candidate web services from a set of functionally equivalent services. In this paper we define a Colored Petri Net (CPN) model which involves transactional properties of web services in the composition process. To ensure reliable and correct execution, unfolding processes of the CPN are followed. The execution of transactional composition Web service (TCWS) is formalized by CPN properties. To identify the best services of QoS properties from candidate service sets formed in the TCSW-CPN, we use skyline computation to retrieve dominant Web service. It can overcome that the reduction of individual scores to an overall similarity leads to significant information loss. We evaluate our approach experimentally using both real and synthetically generated datasets.

## 1. Introduction

Web services are distributed applications that interoperate across heterogeneous networks and that are hosted and executed on remote systems. Service oriented architecture (SOA) is gaining prominence as a key architecture because it allows well-formed and autonomous components to be reused rather than creating new one from scratch. On SOA, web services composition focuses on how to integrate existing web services in diverse and heterogeneous distributed environments, providing different functional, nonfunctional, and behavioral features, to quickly construct workable applications or software for satisfying the requirements which are requested by users and unable to be fulfilled by any single web service.

In order to implement web services composition, component Web services are selected according to user requirements, some constraints and preferences. The selected services usually have the best QoS. However, the interoperation of distributed software systems is always affected by failures, dynamic changes, and availability of resources [1]. The composite web service will not guarantee reliable execution and consistency if the component services are chosen only according to QoS and functional attributes. Transactional properties of selected service should be considered to ensure reliable execution of composite web services. Besides, numerous web services are spread all over Internet, and it is intractable to select appropriate web services satisfying the goal efficiently. Existing various approaches use aggregating parameters and utility function to get score of service. One direction is to assign weights, determined through user feedback, to individual scores [2, 3]. Appropriate weights are chosen either by assuming a priori knowledge about the user's preferences or by applying expensive machine-learning techniques. Both alternatives face serious drawbacks and raise a series of other issues to be solved. More often, these approaches lead to information loss that significantly affects the retrieved results accuracy. For example, use utility function, and finally return the web services with moderate attributes; thus, service with only one bad attribute will be excluded from the result, even though they are potentially good alternatives.

We will use Colored Petri Net as formalism to represent composite web service and perform a Best-First search, where transactional and QoS properties are both integrated in the selection process. But the selection is done in two separate steps, transactional service selection starts firstly, and the QoS-aware service selection is embedded with the transactional-aware service selection [4]. As a tremendous amount of different QoS web services after transactional-aware service selection are spread all over Internet, it is intractable to find the appropriate web services satisfying the given goal quickly. What is more, using traditional methods, services with only one bad QoS attribute may be excluded from the result set, even though they are potentially good alternatives, and thus leads to information loss that significantly affects the retrieved results accuracy. But skyline computation is a nondiscriminating comparison of several numerical attributes at the same time and treats each service equally. We use skyline computation to reduce the number of candidate services and speed up the selection process.

We find that CPN model allows describing not only a static vision of a system, but also its dynamic behavior, and it is expressive enough to capture the semantics of complex web services combinations and their respective interactions. We incorporate transactional web services properties in the CPN model. To ensure reliable and correct execution, unfolding processes of the CPN are followed. The execution of transactional composition web service (TCWS) is formalized by CPN properties. To identify the best services of QoS properties from candidate service sets formed in the TCSW-CPN, we use skyline computation to retrieve dominant web service. It can overcome that the reduction of individual scores to an overall similarity leads to significant information loss. We also define QoS-based dominance relationships between services. To identify the best services from CPN model in QoS properties, we use skyline computation to retrieve dominant web service.

## 2. Related Work

In the last years, although the problem of web service selection and composition has received much attention of many researchers, designing a composite web service which ensures not only correct and reliable execution but also optimal QoS remains an important challenge. Indeed, these two aspects of selection are always implemented separately.

Web services transactions have received much attention recently. Industrial web services transaction specifications emerge. WS-atomic transaction, WS-business activity, and WS-TXM rely on ATM to define transactional coordination protocols. Like ATM these protocols are unable in most cases to model Business process due to their limited control structure. It also ensures reliability on behalf of process adequacy or the opposite. Indeed, a transactional pattern taken alone as a composition of transactional patterns can be considered as a transactional protocol.

In one hand, WSBPEL and WS-CDL follow a workflow approach to define services compositions and services choreographies. Like workflow systems these two languages meet the business process need in terms of control structure. However, they are unable to ensure reliability especially according to the designers' specific needs.

Transaction has achieved a great success in the database community [4, 5]. One of the most important reasons is that the operations in database have clear transactional semantics. However, this is not the case in web services. To solve this problem, the extension mechanism of WSDL can be exploited to explicitly describe the transactional semantics of web services operations [6, 7]. 

There are many works that adopt three kinds of transactional properties proposed in [8] to express the different transactional semantics of web services. Based on this classification, Bhiri et al. [9] analyze the termination property of a composite service. Rusinkiewicz and Sheth [10] define a set of transactional rules to verify the required failure atomicity specified by ATS [11], given the skeleton of a composite service and the transactional properties of its component services. Zeng et al. [12] propose an approach to deduce the required transactional properties of every task based on ATS and then use the result to guide service selection.

For these researches web services composition based on transactional properties ensures a reliable execution; however, an optimal QoS composite web service is not guaranteed.

QoS guarantee for web services is one of the main concerns of the SLA framework. There are projects studying QoS-empowered service selection. In [13], authors present a QoS-aware web service composition which is middleware-supporting quality driven. But the method is based on integer linear programming and best suited for small-size problems as its complexity increases exponentially with the increasing problem size. For [14], the authors propose an extensible QoS computation model that supports an open and fair management of QoS data by incorporating user feedback. However, the problem of QoS-based composition is not addressed by this work. The work of Zeng at al. [15, 16] focuses on dynamic and quality-driven selection of services. The authors use global planning to find the best service components for the composition. They use linear programming techniques [17] to find the optimal selection of component services. Linear programming methods are very effective when the size of the problem is small but suffer from poor scalability due to the exponential time complexity of the applied search algorithms [18]. Despite the significant improvement of these algorithms compared to exact solutions, both algorithms do not scale with respect to the number of candidate web services and hence are not suitable for real-time service composition. The proposed skyline-based algorithm in this paper is complementary to these solutions as it can be used as a preprocessing step to prune noninteresting candidate services and hence reduce the computation time of the applied selection algorithm.

With the above quotation, the approaches implement conventional optimal QoS composition, but composing optimal QoS web services does not guarantee a reliable execution of the resulting composite web service. Therefore, transactional based and QoS based should be integrated.

## 3. A Colored Petri-Net Model of Web Service Composition

Due to the inherent autonomy and heterogeneity of web service it is difficult to predict the overall behavior of a composite service. Unexpected behavior or failure implement of a component service might not only lead to its failure but also may bring negative impact on all the participants of the composition. Web service composition process must satisfy transactional property to provide reliable and consistent execution.

### 3.1. Transactional Property Description

A transactional web service is a web service of which the behavior manifests transactional properties. The main transactional properties of a web service we are considering are pivot, compensatable, and retriable [19]. When transactional property of a service is pivot (*p* for short), the service's effects remain forever and cannot be semantically undone if it completes successfully, and it has no effect at all if it fails. When a service is compensatable (*c* for short), it offers compensation policies to semantically undo its effects. When a service is said to be retriable (*r* for short), it ensures successful completing after several finite activations. Moreover, the transactional property can be combined, and the set of all possible combinations is {*p*, *c*, *pr*, *cr*} [4].

El Haddad et al. [4, 20] extended the previous described transactional properties and adapted them to CWS. A CWS is atomic (*a* for short), if all its component web services complete successfully, they cannot be semantically undone, if one component service cannot complete successfully, previously successful component services have to be compensated. *cs* is compensatable (*c* for short) if all its component services are compensatable. A CWS is retriable (*r* for short), if all its component services are retriable. Transactional composite web service (TCWS) is CWS whose transactional property is in {*a*, *ar*, *c*, *cr*}.

### 3.2. Tolerance Level

In order to provide expression of user transactional criteria, we define tolerance that gives importance of the uncertainty of application completion and recovery for user. A CWS with transactional property *a* or *ar* has greater risk of success completion and recovery than the CWS with transactional property *c* or *cr* [21]. The reason is that properties *a* and *ar* mean once a service has been executed, and it cannot be rolled back. Therefore, we define two levels of tolerance in a transactional system.

*Tolerance 0 *(*T*_0_). The system guarantees that if the execution is successful, the obtained results can be compensated by the user. In this level the selecting process generates a compensatable workflow [4].

*Tolerance 1 *(*T*_1_). The system does not guarantee the successful execution, but if it achieves, the results cannot be compensated by the user. In this level the selecting process generates an atomic workflow [4].

In both tolerance cases, if the execution is not successful, then no result is reflected to the system; nothing is changed on the system.

### 3.3. TCWS-CPN Definition

A colored petri net (CPN) is one of the very useful graphical and mathematical representations, and it has a well-defined semantics for describing states and actions of web service composition. We build a colored petri net model of transactional web service composition (CPN-TWSC). It provides a formalism to depict transactional selections of component services. Besides, functional conditions are expressed as input and output attributes, and transactional properties expressed as a tolerance level. The composite web service will satisfy user's functional requirement and will ensure executing reliably and consistently.

**Definition 1 (TCWS-CPN)**

We define a CPN to transactional composite web services (TCWS-CPN) as a tuple (*P*, *T*, Pre, Post, *C*, *cd*), where

- *P*  is a finite nonempty set of places, with colors in the set *C*. In our case, *P* is composed input and output attributes of web services in the TCWS, functional and transactional requirement, and colors,
- *T* is a finite set of transitions, corresponding to candidate component services execution, *P*∩*T* = *ϕ*,
- *C* is a set of color, which is composed of transactional properties of web services and composition pattern, *C* = *C*_1_ ∪ *C*_2_ = {*p*, *pr*, *a*, *ar*, *c*, *cr*} ∪ {sequence, parallel},
- *cd* : *P* ∪ *T* → *C*. *cd* is a mapping from places or transition set to colors set,
- Pre, Post ∈*β* ^∣*P*∣×∣*T*∣^ are backword incidence matrix and forward incidence matrix of CPN. *β* can be taken as the set of mappings of the form *f* : *cd*(*t*) → Bag(*cd*(*p*)). Pre[*p*, *t*] : *cd*(*t*) → Bag(*cd*(*p*)) and Post[*p*, *t*] : *cd*(*t*) → Bag(*cd*(*p*)) are mappings for each pair (*p*, *t*) ∈ *P* × *T*. Bag(*cd*(*p*)) denotes the set of all multisets over *cd*(*p*). They indicate the input and output execution dependencies during composite web service formation.
  - To denote the places connected to a transition, we use the following notation. *F* is a flow relation *F*⊆(*P* × *T*)∪(*T* × *P*) for the set of arcs. Given an element *x* ∈ *P* ∪ *T*, then ·*x* : = {*y* ∈ *P* ∪ *T* | (*y*, *x*) ∈ *F*} denotes the set of all input elements of *x*, and *x* · : = {*y* ∈ *P* ∪ *T* | (*x*, *y*) ∈ *F*} denotes the set of all output elements of *x*. If *x* is a place, then ·*x* and *x*· denote the set of input and output transitions, respectively.
  - Place is labeled as {*I*, *O*, *I*_*R*_, *O*_*R*_, *T*_*R*_}. In our specific model, a TCWS-CPN will have only initially place *p*_0_, such that *p*_0_ = *ϕ*, which will be initially marked with one token of color. Because it is clear for transactional requirement of user, it will correspond to the only color of transactional property. As color token of every place is transactional property of composite web service, the color set of places is {*a*, *ar*, *c*, *cr*}.
  - Transition includes two basic activities, selecting new component services by means of transactional property and compositing the present component services. Color of transition denotes transactional property of new selecting component services.
  - *cd*(*p*) expresses color of place *p*, and *cd*(*t*) expresses color of transition *t*.
  - Pre[*p*, *t*] ∈ Bag(*cd*(*p*)): there is an arc with arc color from a place *p* ∈ *P* to some transition *t* ∈ *T*, and Post[*p*, *t*] ∈ Bag(*cd*(*p*)): there is an arc from a transition *t* ∈ *T* to some place *p* ∈ *P*. Hence, *F* : = {(*p*, *t*) ∈ *P* × *T* | Pre[*p*, *t*]}∪{(*t*, *p*) ∈ *T* × *P* | Post[*p*, *t*]} is the set of arcs of CPN.

**Definition 2**

A marking of TCWS-CPN = (*P*, *T*, Pre, Post, *C*, *cd*) is a vector *m* such that *m*[*p*] ∈ Bag(*cd*(*p*)) for each *p* ∈ *P*, and *m*[*p*] is component of vector *m* which gives the multiset of color token in place *p*. TCWS-CPN together with a marking *m* is called a TCWS-CPN system and is denoted by *S* = 〈TCWS-CPN, *m*〉. 〈TCWS-CPN, *m*〉 assigns a multiset of colors to each place, which represents the current transactional state of web service composition system.

### 3.4. Services Selection of Transactional Property in the TCWS-CPN

In the section, we focus on web services composition satisfying the user's functional, transactional requirements. We define guard to express transactional restriction of services selection. Binding determines transactional property of selected services. Firing rules are selection rules for component services of transactional property.

**Definition 3 (guard)**

The appropriate restriction is defined by a predicate at the transition which is called a guard. In our TCWS-CPN model, variable “tpattern” is guard of transition, which expressed fired pattern of transition. (That is composition pattern of selected services.)

**Definition 4 (binding)**

A binding is an assignment of values to variables, and variables appear both in the guard of *t* and in the arc expressions of the arcs connected to *t*.

**Definition 5 (firing rules)**

A marking of TCWS-CPN and a binding *B* enable a transition *t* if and only if all its input places contain tokens such that (for  all  *p* ∈ (·*t*), *m*[*p*] ≠ *ϕ*), and at least one of the following conditions is fulfilled:

1. *m* = *m*_0_ (initial marking),
2. (*m*[*p*] ∈ Bag(*a*, *ar*))∧(*B*(*t*pattern) = sequence)∧[*cd*(*t*)∈{*pr*, *ar*, *cr*}],
3. (*m*[*p*] ∈ Bag(*c*, *cr*)∧(*B*(*t*pattern) = sequence)∧[*cd*(*t*)∈{*p*, *a*, *c*, *pr*, *ar*, *cr*}],
4. (*m*[*p*] ∈ Bag(*a*))∧(*B*(*t*pattern) = parallel)∧(*cd*(*t*) = *cr*),
5. (*m*[*p*] ∈ Bag(*ar*))∧(*B*(*t*pattern) = parallel)∧[*cd*(*t*)∈{*pr*, *ar*, *cr*}],
6. (*m*[*p*] ∈ Bag(*c*))∧(*B*(*t*pattern) = parallel)∧[*cd*(*t*)∈{*c*, *cr*}],
7. (*m*[*p*] ∈ Bag(*cr*))∧(*B*(*t*pattern) = parallel)∧[*cd*(*t*)∈{*p*, *a*, *c*, *pr*, *ar*, *cr*}].

**Definition 6 (successor marking relation)**

A successor marking relation is defined by $m\overset{t,B}{\to }m^{\prime }\Leftrightarrow m\geq$pre[·, *t*]*B*∧  *m*′. The *m*′ is obtained after a transition *t* ∈ *T* is fired for binding *B* in a marking *m*.

In our web service composition, a concrete service should be selected only one time and the corresponding transition in TCWS-CPN should be fired only one time. For this reason, when a transition is fired, in the successor marking relation tokens of input places are removed and new tokens are added to the output places. Tokens are added to the output places of transition *t* according to the following rules [20]:

1. if (∃*p*_*i*_ ∈ ( ^·^ *t*) | *a* ∈ *m*_[*p*]_), then (for  all  *p*_*i*+1_ ∈ (*t* ^·^) | *m*_[*p*_*i*+1_]_′) ∈ Bag(*a*),
2. if (∃*p*_*i*_ ∈ ( ^·^ *t*) | *ar* ∈ *m*_[*p*]_), then (for  all  *p*_*i*+1_ ∈ (*t* ^·^) | *m*_[*p*_*i*+1_]_′) ∈ Bag(*ar*),
3. if (∃*p*_*i*_ ∈ ( ^·^ *t*) | *c* ∈ *m*_[*p*]_)∧(*cd*(*t*)∈{*p*, *pr*, *a*, *ar*})∧(*B*(*t*pattern) = sequence), then (for  all  *p*_*i*+1_ ∈ (*t* ^·^) | *m*_[*p*_*i*+1_]_′) ∈ Bag(*a*),
4. if (∃*p*_*i*_ ∈ ( ^·^ *t*) | *c* ∈ *m*_[*p*]_)∧(*cd*(*t*)∈{*c*, *cr*}), then (for  all  *p*_*i*+1_ ∈ (*t* ^·^) | *m*_[*p*_*i*+1_]_′) ∈ Bag(*c*),
5. if (∃*p*_*i*_ ∈ ( ^·^ *t*) | *cr* ∈ *m*_[*p*]_)∧(*cd*(*t*)∈{*pr*, *ar*}), then (for  all  *p*_*i*+1_ ∈ (*t* ^·^) | *m*_[*p*_*i*+1_]_′) ∈ Bag(*ar*),
6. if (∃*p*_*i*_ ∈ ( ^·^ *t*) | *cr* ∈ *m*_[*p*]_)∧(*cd*(*t*)∈{*cr*}), then (for  all  *p*_*i*+1_ ∈ (*t* ^·^) | *m*_[*p*_*i*+1_]_′) ∈ Bag(*cr*),
7. if (∃*p*_*i*_ ∈ ( ^·^ *t*) | *cr* ∈ *m*_[*p*]_)∧(*cd*(*t*)∈{*p*, *a*}), then (for  all  *p*_*i*+1_ ∈ (*t* ^·^) | *m*_[*p*_*i*+1_]_′) ∈ Bag(*a*),
8. if (∃*p*_*i*_ ∈ ( ^·^ *t*) | *cr* ∈ *m*_[*p*]_)∧(*cd*(*t*)∈{*c*}), then (for  all  *p*_*i*+1_ ∈ (*t* ^·^) | *m*_[*p*_*i*+1_]_′) ∈ Bag(*c*).

### 3.5. Composite Sequence in the CPN

**Definition 7 (occurrence sequence)**

We define the set OCC(S) of occurrence sequences to be the set of all sequences of the form *m*_0_, *t*_0_, *m*_1_, *t*_1_, *m*_2_, *t*_2_,…, *t*_*n*−1_, *m*_*n*_  (*n*⩾1) such that $m_{i}\overset{t_{i}}{\to }m_{i+1}$ for *i* ∈ {0,…, *n* − 1}.

Occurrence sequence in fact represents the selection of several web services, *s*_0_ ⋯ *s*_*n*−1_, which are components of the resulting TCWS, whose aggregated TP is *m*_*n*_.

**Definition 8 (reachability set)**

For a TCWS-CPN system *S* = 〈TCWS-CPN, *m*〉 the set $\text{RS}(S)=\text{RS}(\text{TCWS-CPN},m):=\{m|\exists w\in T^{*}\cdot m_{0}\overset{w}{\to }m\}$ is the reachability set.

One of the goals in my paper is discovering and selecting the web services whose composition satisfies the functional and the transactional requirements of the user such as follows.

Our problem consists in discovering and selecting the web services of the registry whose composition satisfies the functional, QoS, and the transactional requirements of the user, which ensures reliable execution of composite web servic such as follows.

**Definition 9 (transactional composite web services problem)**

Given a user query *Q* (it is used to discover component services) and a TCWS-CPN, transactional composite web services problem consists in creating a TCWS-CPN by firing rule of a marking and binding, from the occurrence sequence and reachability set, such that $m_{0}\overset{w}{\to }m_{F}$, where *m*_0_ is the initial marking, *m*_*F*_ is a reachable marking such that: if *T*_*Q*_ = *T*_0_, then for  all  *p* ∈ (*P*∩*O*_*Q*_) and *m*_*F*_[*p*]∈{*c*, *cr*} and if *T*_*Q*_ = *T*_1_, then for  all  *p* ∈ (*P*∩*O*_*Q*_) and *m*_*F*_[*p*]∈{*a*, *ar*, *c*, *cr*}, and such that the composition of all the web services corresponding to the transitions of w represents a TCWS.

## 4. Execution Framework Architecture of TCWS-CPN

### 4.1. Execution Framework Architecture

During the TCWS component web services exist two composition patterns. In sequential patterns, the results of previous services are inputs of successor services which cannot be invoked until previous services have finished. In parallel scenario, several branch services are executed simultaneously because they do not have data flow dependencies. Hence, to ensure that sequential and parallel execution of TCWS satisfies transactional requirement of user, it is mandatory to follow TCWS-CPN model taken by the composer.

**Figure 1:**
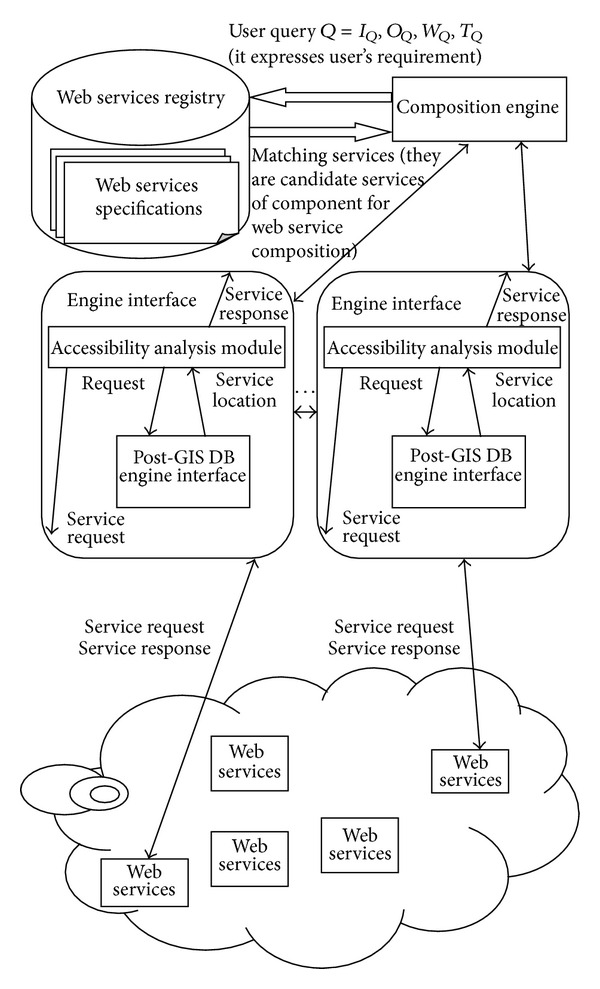
Execution framework architecture.

In this paper, we propose execution framework architecture of TCWS-CPN, in which a Composition Engine manages selection and execution of a TCWS. It is in turn a collection of Composition Threads that is assigned to each Wed service in the TCWS.  Figure 1 depicts the overall architecture of our Executor. The Composition Engine and its Engine Threads are in charge of initiating, controlling, and monitoring the execution, as well as collaborating with its peers to deploy the TCWS execution. The Composition Engine and its Engine Threads are in charge of initiating, controlling, and monitoring the execution, as well as collaborating with its peers to deploy the TCWS execution. The Composition Engine is responsible for initiating the Engine Threads and the TCWS-CPN system, and then Engine Threads are responsible for the invocation of web services, monitoring its execution, and forwarding results to its peers to continue the execution flow. In the framework, all of components are recovery.

The model of proposed framework can distribute the responsibility of executing a TCWS across several Engine Threads, which is implemented in a distributed memory environment supported by message passing or in a shared memory platform. The logic of Executor can distribute execution and is independent of implementation, which is place in different physical nodes from those where actual web services are placed. The Composition Engine needs to have access to the web services Registry, which contains the WSDL and OWLS documents. Engine Threads invoke the component web services remotely from web services Registry. The information needed at runtime by each Engine Thread is extracted from the TCSW-CPN in a shared memory implementation or sent by the composition Engine in a distributed implementation.

Generally, the component web services are categorized into two types, atomic and composite web services. An atomic web service invokes local operations. A composite web service accesses additionally other web services or invokes operations of other web services. Transitions in the TCWS-CPN, representing the TCWS, could be atomic web services or TCWS. Atomic web services have its corresponding WSDL and OWLS documents. TCWS can be encapsulated into an executor. The Composition Engine also has its corresponding WSDL and OWLS documents. 

### 4.2. Example

**Figure 2:**
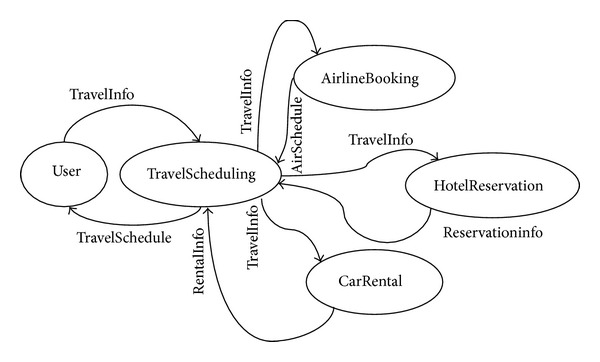
Illustrative state diagrams for travel scheduling.

The example in this paper is based upon a travel-scheduling service composition which is depicted by state diagram in Figure 2. Basic inputs and outputs of candidate service sets which correspond to component services assigned to transitions are shown in Table 1.

**Figure 3:**
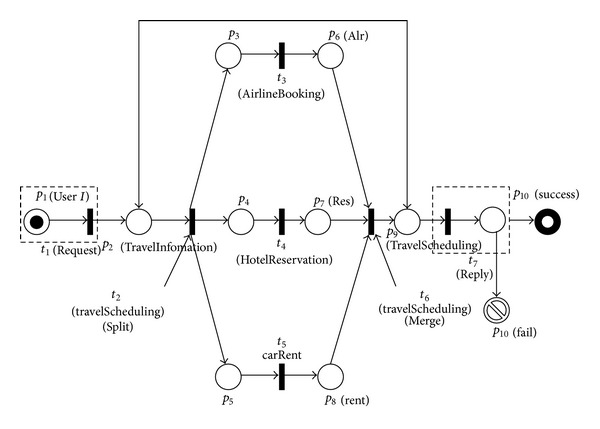
TCWS-CPN for travel scheduling.

Let *I*_*Q*_ = {UserRequest}, *O*_*Q*_ = {User  TravelPlan}, *T*_*Q*_ = *T*_1_, *m*_0_ = (*pr*, *ϕ*, *ϕ*, *ϕ*, *ϕ*, *ϕ*, *ϕ*, *ϕ*, *ϕ*, *ϕ*). According to *I* and *I*_*Q*_, place *p*_1_ is created. The set of Candidate services for *p*_1_ is also formed by query from registry. In order to satisfy transactional request transition *t*_1_ is added to TCWS-CPN based on *m*_0_ and firing rule (1), and token of *t*_1_ is *cd*(*t*_1_)∈{*a*, *ar*, *c*, *cr*}. As web service of user request is retriable shown in Figure 3, let *cd*(*t*_1_) = *ar*. Meanwhile candidate services of *t*_1_ are pruned, which are kept with transactional property *cr* and deleted with other transactional properties. Then an arc is created from *p*_1_ to *t*_1_. One of candidate services is assigned to transition *t*_1_ and takes part in web service composition. Place of *p*_2_ is created after *t*_1_ is fired, and pre[·, *t*_1_] of the arc is also created. Rule of successor marking relation enables *m*_1_ = (*ϕ*, *ar*, *ϕ*, *ϕ*, *ϕ*, *ϕ*, *ϕ*, *ϕ*, *ϕ*, *ϕ*). Generating the rest parts of TCWS-CPN, including marking of places, token and binding of transitions, and backward and forward matrix of arcs is shown as (1). Marking of places and token and binding of transitions are expressed in occurrence sequence (Definition 8):

(1) m  o  =(prϕϕϕϕϕϕϕϕϕ)→t1(cd(t1)=pr∧B(tparttern)=sequence)m  1  =(ϕarϕϕϕϕϕϕϕϕ)→t2(cd(t2)=cr∧B(tparttern)=sequence){m  2  =(ϕϕarararϕϕϕϕϕ)→t3(cd(t3)=ar∧B(tparttern)=parallel)m  5  =(ϕϕϕϕϕarararϕϕ)m  3  =(ϕϕarararϕϕϕϕϕ)→t4(cd(t4)=cr∧B(tparttern)=parallel)m6=(ϕϕϕϕϕarararϕϕ)m  4  =(ϕϕarararϕϕϕϕϕ)→t5(cd(t5)=cr∧B(tparttern)=parallel)m  7  =(ϕϕϕϕϕarararϕϕ)}→t5(cd(t5)=pr∧B(tparttern)=sequence)m  8  =(ϕϕϕϕϕϕϕϕarϕ)→t6(cd(t6)=cr∧B(tparttern)=sequence)m  9  =(ϕϕϕϕϕϕϕϕϕar).

## 5. Qos-Based Skyline Web Services

### 5.1. The Skyline Computation Problem

The basic skyline consists of all nondominated database objects. That means all database objects for which there is no object in the database that is better or equal in all dimensions, but in at least one aspect strictly better. Assuming every database object to be represented by a point in *n*-dimensional space with the coordinates for each dimension given by its scores for the respective aspect, we can formulate the problem as follows.

*The Skyline Problem*. Given set *O* : = {*o*_1_,…, *o*_*N*_} of *N* database objects, *n* score functions *s*_1_,…, *s*_*n*_ with *s*_*i*_ : *O* → [0,1] and *n* sorted lists *S*_1_,…, *S*_*n*_ containing all database objects and their respective score values using one of the score function is for each list; all lists are sorted descending by score values starting with the highest scores. Wanted is the subset *P* of all non-dominated objects in *O*, that is, {*o*_*i*_ ∈ *P* | ¬∃*o*_*j*_ ∈ *O* : (*s*_1_(*o*_*i*_) ⩽ *s*_1_(*o*_*j*_)∧⋯∧*s*_*n*_(*o*_*i*_) ⩽ *s*_*n*_(*o*_*j*_)∧∃*q* ∈ [1,…, *n*] : *s*_*q*_(*o*_*i*_) < *s*_*q*_(*o*_*j*_))}.

### 5.2. Skyline Web Services for Qos-Based Composition

QoS-based service composition is a constraint optimization problem which aims at selecting individual services that meet QoS constraints and also provide the best value for the utility. For a composite web service with *n* activities and *l* candidate services per activity, there are *l* ^*n*^ possible combinations to be examined. Hence, performing an exhaustive search can be very expensive in terms of computation time and, therefore, inappropriate for run-time service selection in applications with many services and dynamic needs. Skyline computation offers a new solution of finding optimal data from huge data sets, whose computation can be expensive and whose applications require fast response times.

The main idea in our approach is to perform a skyline query on the services in each activity to distinguish between those services that are potential candidates for the composition and those that cannot possibly be part of the final solution. The latter can effectively be pruned to reduce the search space.

**Definition 10 (dominance)**

Given a service set *S*_*A*_*i*__ assigned to activity *A*_*i*_ having *n* candidate services: *S*_*A*_*i*1__,  *S*_*A*_*il*__,…, *S*_*A*_*in*__, QoS vector is d dimensions: *q*_1_(*S*_*A*_*il*__), *q*_2_(*S*_*A*_*il*__),…, *q*_*d*_(*S*_*A*_*il*__). *S*_*A*_*iu*__ is said to dominate *S*_*A*_*iv*__, denoted as *S*_*A*_*iu*__≺*S*_*A*_*iv*__ if and only if *S*_*A*_*iu*__ is better than or equal to *S*_*A*_*iv*__ in all attributes and strictly better in at least one attribute, that is, for  all  *k* ∈ [1, *d*] : *q*_*k*_(*S*_*A*_*iu*__) ≤ *q*_*k*_(*S*_*A*_*iv*__) and ∃*l* ∈ [1, *d*] : *q*_*k*_(*S*_*A*_*iu*__) < *q*_*k*_(*S*_*A*_*iv*__).

If *S*_*A*_*iv*__ is neither dominated by nor dominates *S*_*A*_*iu*__, then *S*_*A*_*iv*__ and *S*_*A*_*iu*__ are incomparable. The notion of dominance handles requirement since comparing between matched services takes into consideration the degrees of match in all parameters, instead of calculating and using a single, overall score.

**Definition 11 (skyline web services [22])**

The skyline web services of a service set *S*_*Ai*_, denoted by SWS, comprise the set of those services that are not dominated by any other services, that is, SWS = {*S*_*A*_*iu*__ ∈ *S*_*A*_*i*__ | ¬∃*S*_*A*_*iv*__ ∈ *S*_*A*_*i*__ : *S*_*A*_*iv*__≺*S*_*A*_*iu*__}. Services in SWS are skyline web services of a service set *S*_*A*_*i*__.

**Figure 4:**
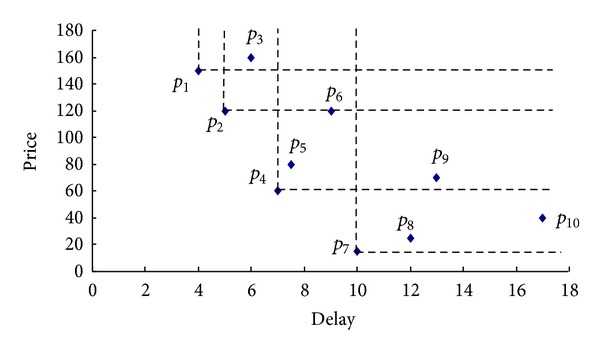
Skyline Services.

We observe that only those services that belong to the SWS are not dominated by any other functionally equivalent service and are valid candidates for the composition. This provides a valid pruning of the number of candidate services.  Figure 4 shows an example of skyline services of candidate services for a certain activity. Each service is described by two QoS attributes, namely, delay and price. Hence, the services are represented as points in the 2-dimensional space, with the coordinates of each point corresponding to the values of the service in these two parameters. SWS includes four elements, SWS = {*p*_1_, *p*_2_, *p*_4_, *p*_7_}, because they are not dominated by any other service. On the other hand, service *p*_6_ is not contained in the SWS because it is dominated by the services *p*_2_ and *p*_4_. 

The skyline web services provide different tradeoffs between the QoS attributes and are incomparable to each other, as long as there is no prespecified preference scheme regarding the relative importance of these attributes. For example, for a specific user, a service may be the most suitable choice, due to its very low delay and despite its high price, while for the other user, a service may be the most preferred one due to its low price.

### 5.3. Skyline Algorithm of Qos-Based Web Service Selection

 (0) Initialize a data structure SWS : = Φ containing records with an identifier and *n* real values indexed by the identifiers, initialize *n* lists *K*_1_,…, *K*_*n*_ : = Φ containing records with an identifier and a real value, and initialize *n* real values *p*_1_,…, *p*_*n*_ : = 1.
 
(1) Initialize counter *i* : = 1.
 (2) Get the next object *S*_*A*_*i*new__ by sorted access on list *S*_*A*_*i*__.
 (3) If *S*_*A*_*i*new__ ∈ SWS, update its record's *i*th real value with *S*_*A*_*i*__(*S*_*A*_*i*new__), else create such a record in SWS.
 (4) Append *S*_*A*_*i*new__ with *S*_*A*_*i*__(*S*_*A*_*i*new__) to list *K*_*i*_.
 (5) Set *p*_*i*_ : = *S*_*A*_*i*new__ and *i* : = (*i*mod⁡*n*) + 1.
 (6) If all scores *S*_*A*_*i*__(*S*_*A*_*i*new__)  (1 ≤ *i* ≤ *n*) are known, proceed with Step (2) else with Step(4)
 (7) For *i* = 1 to *n* do.
 (8) While *p*_*i*_ = *S*_*A*_*i*__(*S*_*A*_*i*new__) do sorted access on list *S*_*A*_*i*__ and handle the retrieved objects like in Step (2) to (3).
 (9) If more than one object is entirely known, compare pairwise and remove the dominated objects from SWS.
 (10) For *i* = 1 to *n* do.
 (11) Do all necessary random accesses for the objects in *K*_*i*_ that are also in SWS, and immediately discard objects that are not in SWS.
 (12) Take the objects of *K*_*i*_ and compare them pairwise with the objects, in *K*_*i*_. If an object is dominated by another object remove it from *K*_*i*_ and SWS.
 (13) Output SWS as the set of skyline web services.

## 6. Experimentation

In the section, we use two scenarios to evaluate the behavior of TCWS-CPN and the efficiency of our approach. We have conducted experiments in two scenarios. The first scenario: different services are generated to verify the validity of our TCWS-CPN model. In the second one, we use the OWL-S service retrieval test collection OWLS-TC v2^2^. The execution time of QoS services selection with skyline computation is compared with that without skyline computation.

The first scenario is implemented as follows. In order to evaluate the behavior of transactional selection approach based on TCWS-CPN, 10 user queries are generating with various kinds of inputs and outputs. The services are generating randomly from an ontology containing 20 generated elements each of which has between 1 and 5 inputs and between 1 and 3 outputs [20]. Every generated service is independently from the others. Experiments are conducted by implementing the proposed service selection approach with the program on a PC Core i3 with 2 GB RAM, Windows 7, and Java 2 Enterprise Edition V1.5.0. JDK 1.6 virtual machine is used to develop and run the program. The experiments involved composite services varying the number of activities and varying the number of web services. 

In the first scenario, every web service has between 1 and 5 inputs and outputs, randomly generated from an ontology containing 10 generated elements. QoS attribute and transactional property of every service are also generated randomly but there are the relations between them. To model the fact, we assume that the execution price of service whose transactional property is *c* is more expensive than whose transactional property is *p* or *a*, because the former provides additional functionality in order to guarantee that the result can be compensatable. Similarly, we believe that a *pr*, *ar*, or *cr* web service has execution duration higher than a nonretriable one, because the former provides additional operation in order to guarantee that it successfully finishes after a finite number of invocations. In addition, user's requirement and QoS weight have been randomly generated by varying number of inputs and outputs between 1 and 3.

Relationship of utility value and duration weight with different tolerance are depicted the more important the duration criteria to the user, the better a composition with tolerance *T*_0_ compared to a composition with tolerance *T*_1_.

**Figure 5:**
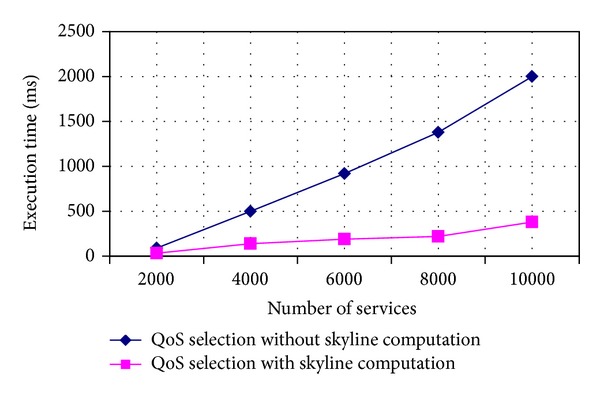
Execution time.

For the second scenario, we use the OWL-S service retrieval test collection OWLS-TC v2^2^. This collection contains services retrieved mainly from public IBM UDDI registries and semiautomatically transformed from WSDL to OWL-S. We apply skyline to select the best candidates for QoS selection. We compare execution time of QoS selection using skyline computation with the time without using Figure 5 illustrates the running time of QoS selection with (and without) skyline computation. Observe that the time without using skyline computation is higher than that using it.

## 7. Conclusion

CPN model allows describing not only a static vision of a system, but also its dynamic behavior, and it is expressive enough to capture the semantics of complex web services combinations and their respective interactions. In the paper we propose a hybrid solution that takes advantage of search metaheuristics techniques to consider functional conditions expressed as input and output attributes, and transactional properties expressed as a tolerance level. We incorporate transactional web services properties in the CPN model. To ensure reliable and correct execution, unfolding processes of the CPN are followed. The execution of transactional composition web service (TCWS) is formalized by CPN properties. To identify the best services of QoS properties from candidate service sets formed in the TCSW-CPN, we use skyline computation to retrieve dominant web service. It can overcome that the reduction of individual scores to an overall similarity lead to significant information loss. We also define QoS-based dominance relationships between services. To identify the best services from CPN model in QoS properties, we use skyline computation to retrieve dominant web service. We have shown how the best matches can be identified efficiently by a skyline computation algorithm, and we have addressed common tasks involved in the service selection process, referring both to the requesters' and the providers' perspectives.

In the experimentation, our intention is to compare both implementations under different characterizations of CPNs. Experimental evaluation on real and synthetic data shows that the best matches can be identified very efficiently, with a significant increase in recall and precision.

At the same time, the next step is to add automatic data mapping functionality into our system, using the semantic-based approach. In this paper, we utilize product-specific property to facilitate mediator service generation. To improve our prototype of the service selection process, by facilitating the user in expressing and refining his/her queries and providing faceted browsing capabilities.

## Figures and Tables

**Table 1:** Example.

Service class name	Input	Output
s1	UserRequest	TravelScheduling
s2	TravelScheduling	AirlineRequest, HotelRequest, CarRentRequest
s3	AirlineRequest	AirlineScheduling
s4	HotelRequest	HotelScheduling
s5	CarRentRequest	CarRentScheduling
s6	AirlineScheduling, HotelScheduling, CarRentScheduling	TravelPlan
s7	TravelPlan	User TravelPlan
